# Physiology of Layer 5 Pyramidal Neurons in Mouse Primary Visual Cortex: Coincidence Detection through Bursting

**DOI:** 10.1371/journal.pcbi.1004090

**Published:** 2015-03-13

**Authors:** Adam S. Shai, Costas A. Anastassiou, Matthew E. Larkum, Christof Koch

**Affiliations:** 1 California Institute of Technology, Pasadena, California, United States of America; 2 Allen Institute for Brain Science, Seattle, Washington, United States of America; 3 Neurocure Cluster of Excellence, Department of Biology, Humboldt University of Berlin, Berlin, Germany; Indiana University, UNITED STATES

## Abstract

L5 pyramidal neurons are the only neocortical cell type with dendrites reaching all six layers of cortex, casting them as one of the main integrators in the cortical column. What is the nature and mode of computation performed in mouse primary visual cortex (V1) given the physiology of L5 pyramidal neurons? First, we experimentally establish active properties of the dendrites of L5 pyramidal neurons of mouse V1 using patch-clamp recordings. Using a detailed multi-compartmental model, we show this physiological setup to be well suited for coincidence detection between basal and apical tuft inputs by controlling the frequency of spike output. We further show how direct inhibition of calcium channels in the dendrites modulates such coincidence detection. To establish the singe-cell computation that this biophysics supports, we show that the combination of frequency-modulation of somatic output by tuft input and (simulated) calcium-channel blockage functionally acts as a composite sigmoidal function. Finally, we explore how this computation provides a mechanism whereby dendritic spiking contributes to orientation tuning in pyramidal neurons.

## Author Summary

Neurons in the brain have elaborate dendritic morphologies, hosting a variety of nonlinear channels that give way to single cell computation. In this study, we perform patch clamp recordings in the apical dendrites to establish the spatial distribution of nonlinear channels and the signals they support in the dendrites of layer 5 pyramidal neurons of the mouse primary visual cortex. Using this data, we create a detailed single cell model and simulate synaptic input. We then summarize the results of the simulations using a simple abstracted model, that ultimately describes the computation layer 5 pyramidal neurons perform on synaptic input. We find that this computation is a form of nonlinear frequency-modulation that works in a dendritic-spike dependent manner. Finally, we show how this computation allows dendritic spikes to contribute to the orientation tuning of pyramidal neurons in the visual cortex.

## Introduction

Layer 5 (L5) of neocortex contains excitatory pyramidal neurons considered a main integration unit of the cortical column that project to other areas of neocortex as well as subcortical structures. These cells uniquely possess dendrites spanning all cortical layers and receive both long-range excitatory and local excitatory and inhibitory inputs [1]. The properties of L5 pyramidal neuron dendrites have been extensively studied *in vitro*, with the vast majority of the electrophysiological work performed in hippocampus [2], somatosensory [3] and prefrontal cortex [4,5] of rats. This line of research has shown that pyramidal neurons in different cortical regions contain voltage-gated Na^+^ channels along the dendritic trunk which support the backpropagation of action potentials (APs) from the soma into dendrites [6], as well as voltage-gated Ca^2+^ channels that support spiking in the apical dendrite [3,7].

Alongside single-cell electrophysiology, connectivity in visual cortex has also been studied in a variety of mammals, including rodents [8,9,10]. Neurons from higher order visual areas, such as the latero-medial area of mouse visual cortex, project axons to the upper layers of the primary visual cortex [11,12]. Alongside these long-range axons, reciprocal excitatory connections also exist [1]. The question of what functional role axons that synapse onto the remote apical dendrites of pyramidal neurons play at the physiological and behavioral level remains largely unanswered. Nevertheless, it is clear these axons target the apical tuft dendrites of pyramidal neurons [13]. Moreover, dendrites of pyramidal neurons in other brain areas are highly electrogenic and heavily influence spiking output [2,3]. How does the aforementioned contribute to visual processing in cortex? To answer this question we use *in vitro* whole-cell patch clamp experiments of both somas and dendrites with a particular interest in measuring the intrinsic electrophysiological properties of apical dendrites, precisely where feedback axons terminate [11]. We find that mouse L5 pyramidal neurons in V1 support backpropagating action potentials (bAPs), and dendritic Ca^2+^ spiking.

What computation might such an electrophysiological setup perform? We use a multi-compartmental model of a L5 pyramidal neuron that replicates the important nonlinearities found in our experiment to establish a coincidence detection algorithm. We show how concurrent input into the perisomatic and electrically remote apical tuft regions switches somatic action potential output from low to high frequency. Thus, high frequency bursting indicates coincident input into different parts of the dendritic structure. We further explore regulation of this coincidence detection scheme by blockage of the voltage-gated calcium channels on which this computational principle depends on. We ultimately show that such single-cell computation can be conceptualized as a composite of sigmoid functions. Finally, we discuss how such a coincidence detector can act as a mechanism whereby dendritic nonlinearities play a role in the sharpness and reliability of orientation tuning in pyramidal neurons.

## Results

We investigate the electrophysiological properties of single L5 pyramidal neurons in area V1 in adult mice (P35-62) by whole cell patch clamping the somata and the axial dendrites to establish a multi-compartmental model. The model is then used to explore the biophysical computation that single neurons perform on spatio-temporal patterns of synaptic input, which can be precisely controlled *in silico* alongside details of the intrinsic biophysics. The cell bodies are located at cortical depth (mean +/- std) 617 +/- 45 μm (range: 460–715 μm) below the pia. In experiments with dendritic patching, electrodes are placed between 72 and 497 μm from the soma with a mean of 270 +/- 149 μm (n = 13) between the soma and the first bifurcation point along the apical dendrite.

### Intrinsic electrogenesis in the dendrites

**Fig 1:**
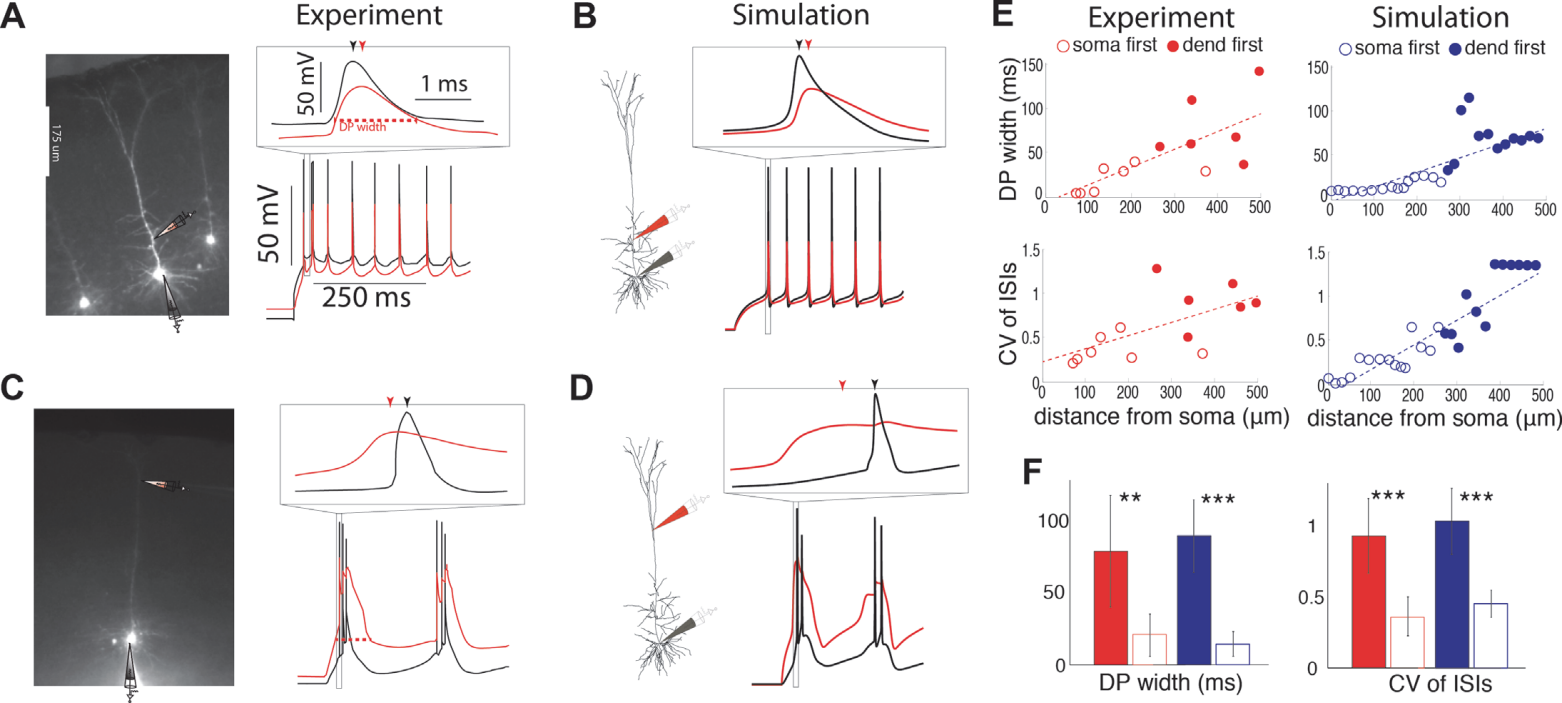
Current injections into the far apical dendrites elicit dendritic electrogenesis. **(a,b,c,d)** Examples of 1 second DC current injections into the dendrites of a L5 pyramidal neuron in V1 in experiments and computer simulations. Red traces show dendritic and black traces show somatic membrane potentials. Insets show details of individual action potentials and dendritic responses. **(a,b)** Membrane response to dendritic current injections at 135 μm from the soma show somatic spiking with relatively constant ISIs giving rise to bAPs in the dendrite. **(c,d)** Membrane response to dendritic current injections 442 μm from the soma show burst firing at the soma and dendritic electrogenesis that precedes action potential firing at the soma. Like in experiments (a,c), in simulations (b,d), injections close to the soma give rise to APs at a regular frequency that backpropagate, while injections farther into the apical dendrite give rise to large dendritic potentials that precede bursts of APs. **(e)** Dendritic potential width (illustrated as red dotted lines in (a,c)) and ISI coefficient of variations as a function of distance of the dendritic current injection from the soma in experiment and simulation. Filled circles corresponds to cases where dendritic spikes precede the somatic spike (e.g. inset *c*,*d*), while open circles correspond to cases where somatic spikes precede the dendritic event (e.g. inset *a*,*b*). Red and blue circles denote experiment and simulation results respectively. Lines are linear fits. **(f)** Comparison of the DP width and coefficient of variation of the ISIs of cases where somatic spiking preceded dendritic events (open bars) and where dendritic electrogenesis preceded somatic spiking (filled bars). Experimental data is in red and simulation data is in blue. *, **, *** Indicate significant differences between the two bars.

Injections of DC current through dendritic pipettes can trigger somatic spiking and bAPs (Fig. 1A), and can, in some cases, induce nonlinear dendritic electrogenesis that precedes somatic spiking (Fig. 1C). A multi-compartmental model can recreate these patterns (Fig. 1BD, see Methods for details). 1 s long dendritic current injections (n = 13) elicit trains of 18.38 +/- 8.52 spikes at the soma. The width of the largest dendritic potential (DP) increases when current is injected farther from the soma (Fig. 1E top left, slope = 0.20 ms μm^-1^, slope is significantly different from zero with p = 0.0043). This is also the case for the coefficient of variation (CV) of the interspike intervals (ISIs) (Fig. 1E bottom left, slope = 0.0015 μm^-1^, p = 0.02), and the ratio of voltage threshold for triggering somatic action potentials between dendritic and somatic step current injection (slope = 0.0040 μm^-1^, p = 3.8e-4). These trends can be recreated well using a multicompartmental model (Fig. 1E right side, see Methods), and is dependent on a calcium “hot-spot” in the apical dendrites of the model. Importantly, both the DP width and the CV of ISIs for the dendrite-first cases (i.e. when dendritic electrogenesis precedes somatic spiking, Fig. 1CD inset; compare to the soma-first case e.g. Fig. 1AB, inset.) are significantly different than in the soma-first cases (Fig. 1F, p = 0.0039 and 4.4e-4 respectively in experiment). Additionally, the dendrite-first cases occur at significantly different distances from the soma than the soma-first cases (p = 0.0016), suggesting that the occurrence of long dendritic potentials and variable somatic ISIs occurs during dendritic injections farther away from the soma.

**Fig 2:**
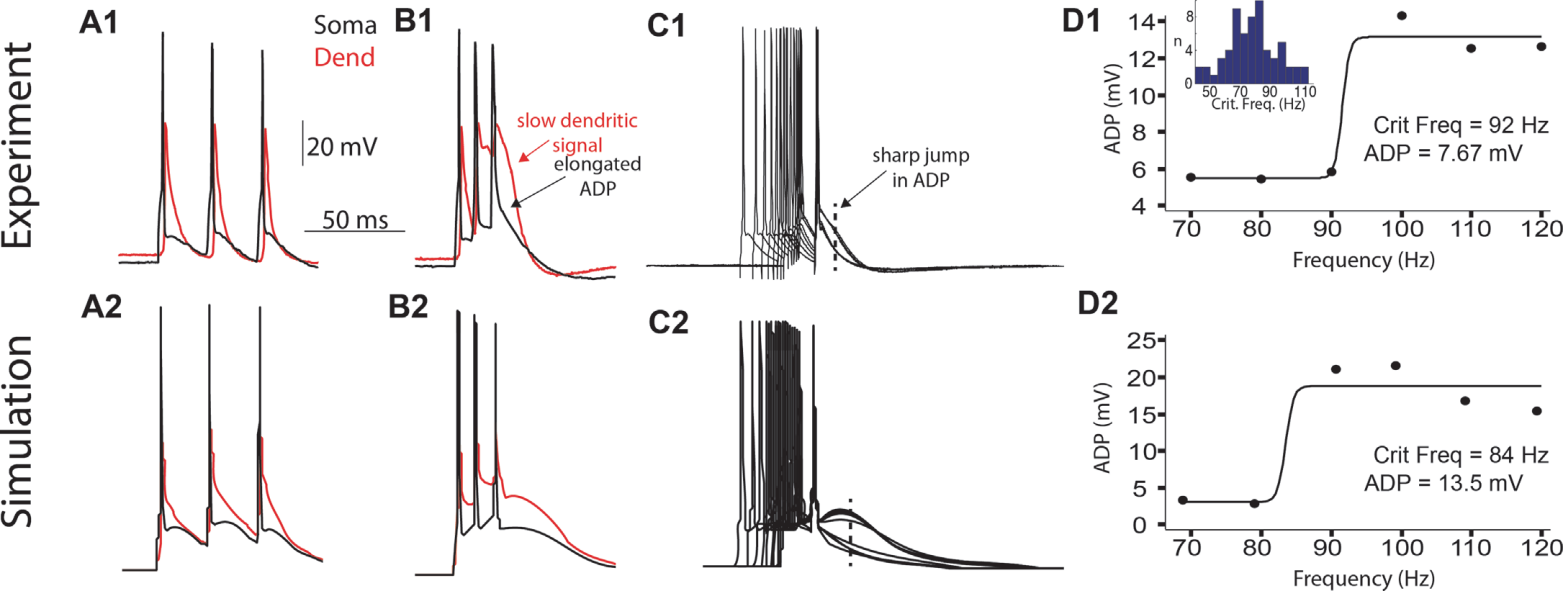
Calcium spiking in the dendrites in response to bursts of action potentials. **(a)** The somatic (black) and dendritic (red) response to three short current pulses at the soma at a slow frequency (70 Hz). Analysis of experimental and simulation data are given in rows 1 and 2 respectively. **(b)** As in (a) except for three pulses above the critical frequency (100 Hz). Note the slow dendritic signal following the last somatic spike as well as the elongated somatic afterdepolarization (ADP) compared to (a). **(c)** Ten somatic responses to increasing frequencies of 3 short DC current injections at the soma, aligned at the final AP. Note the sharp nonlinear jump in ADP shape (broken line) **(d)** ADP size shown at the time of the dotted line in (c) as a function of frequency. The critical frequency is defined as the inflection point of the sigmoidal fit, and ADP size is defined as the difference between the two plateaus. The inset in d1 shows a histogram of the critical frequency for all 66 cells. Simulation results of the critical frequency analysis are shown in the bottom row.

Previous work has shown that bursts of bAPs cause calcium channel-dependent spiking in the dendrites of L2/3, L5, and L6 pyramidal neurons in rat somatosensory cortex [14,15,16]. Here we use the critical frequencies method to detect the presence of a calcium spike hotzone in the dendrites of mouse V1 L5 pyramidal neurons [14], and to fit our model to realistic dendritic behavior. We administer 3 short (2 ms each) current pulses at increasing frequencies (between 10 and 200 Hz, intervals of 10 Hz) at the soma. By aligning the somatic responses to the last AP, a nonlinear increase in the amplitude of the after-depolarization (ADP) is observed (Fig. 2ABC). The frequency at which the nonlinearity in ADP occurs, called *critical frequency*, is indicative of a calcium spike in the apical dendrites [14], can be measured explicitly with a dendritic patch (Fig. 2B), and is also seen in our final multi-compartmental model. ADP at the soma is 4.4 +/- 3.2 mV greater at critical frequencies than lower frequencies, and the critical frequency is 89.7 +/- 17.1 Hz (Fig. 2D). The sharp change in ADP suggests nonlinear recruitment of dendritic current, and the ADP at the soma is clearly dependent on dendritic electrogenesis, since a dendritic nonlinearity occurs for similar critical frequency in all experiments with a dendritic patch. This critical frequency behavior is reproduced in the multicompartmental model (Fig. 2 bottom row), which has a critical frequency of 84 Hz. Since the critical frequency experiment depends on action potential generation perisomatically, active backpropagation of action potentials along the apical axis, calcium-dependent nonlinearities in the apical tuft, and the transfer of nonlinear electrogenesis from the tufts back to the somatic electrode, the model fits both somatic and dendritic properties of L5 pyramidal neurons in mouse V1 well.

### Synaptic inputs in the multicompartmental model

We use the multi-compartmental pyramidal cell model [17] to further explore the relationship between the different nonlinearities found in our experiments and their role in the transformation between synaptic input and action potential output. Our final model and parameter set captures the membrane response to suprathreshold somatic and dendritic current injections (Fig. 1), and the Ca^2+^ spike caused by a critical frequency of somatic APs (Fig. 2). In addition, the model captured both subthreshold and NMDA spiking dynamics (S2 and S3 Figs.). In the simulations we ask how much input is needed into the dendritic tuft in order to elicit a burst of somatic action potentials given a certain amount of basal input. Unlike our experiments, we have complete control over every aspect of the simulation (including synaptic input), and can explicitly study the role of specific conductances (e.g. the Ca^2+^ conductances) in the simulations.

**Fig 3:**
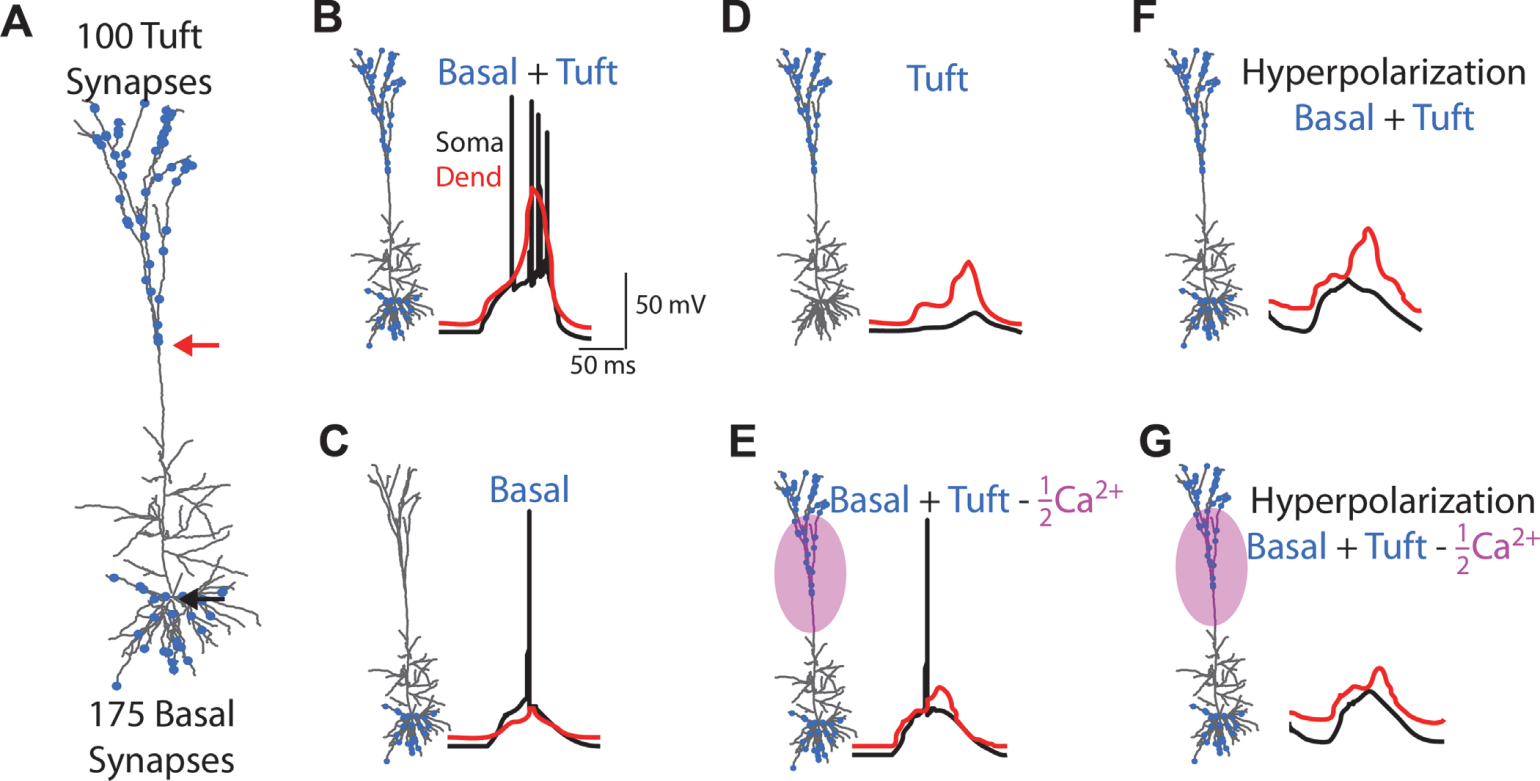
Coincidence detection between basal and apical tuft inputs. **(a)** 100 tuft and 175 basal NMDA/AMPA synapses are distributed randomly across the apical tuft and basal dendrites of a multi-compartmental L5 pyramidal neuron model. All synapses are randomly and uniformly elicited in time across 100 ms. In the following, somatic traces are in black and dendritic (location shown by the red arrow in a), are in red. **(b)** Simultaneous tuft and basal inputs triggers a burst of somatic APs and a dendritic Ca^2+^ spike, while **(c)** basal inputs alone evoke only a single somatic spike. **(d)** Apical tuft inputs alone do not evoke somatic spiking. **(e)** Reducing Ca^2+^ channel conductance by 50% during tuft and basal input gives rise to a single somatic spike. **(f)** When applying a 200 pA hyperpolarizing DC current to the soma, the subthreshold response of the tuft and basal inputs are similar to the case with Ca^2+^ conductances reduced shown in **(g)**, even though the suprathreshold (b,c) cases are remarkably different.

We randomly distribute NMDA/AMPA synapses across the tuft and basal dendrites (Fig. 3A). 100 tuft and 175 basal synapses of equal postsynaptic conductance are elicited randomly and uniformly (in time) within 100 ms. When basal and tuft inputs impinge along the neuron (Fig. 3B), a high frequency burst of 4 somatic (black trace) APs and 1 dendritic (red trace) spike occur. Basal input alone (Fig. 3C) causes a single somatic AP and no dendritic spike, while tuft input alone (Fig. 3D) causes no spiking. Thus, the neuron acts as a coincidence detector for basal and tuft input, signaling coincident input by a high frequency burst. When the conductance of both high- and low- threshold Ca^2+^ channels in the apical dendrites is halved (Fig. 3E), somatic output from basal and tuft input reverts from bursting back to a single AP. To visualize the subthreshold responses to the input we inject 200 pA of hyperpolarizing DC current at the soma. The somatic subthreshold response to basal and tuft input both with and without the reduction in apical Ca^2+^ conductance (Fig. 3FG) is similar, though the dendritic response is slightly smaller when Ca^2+^ conductances are reduced.

**Fig 4:**
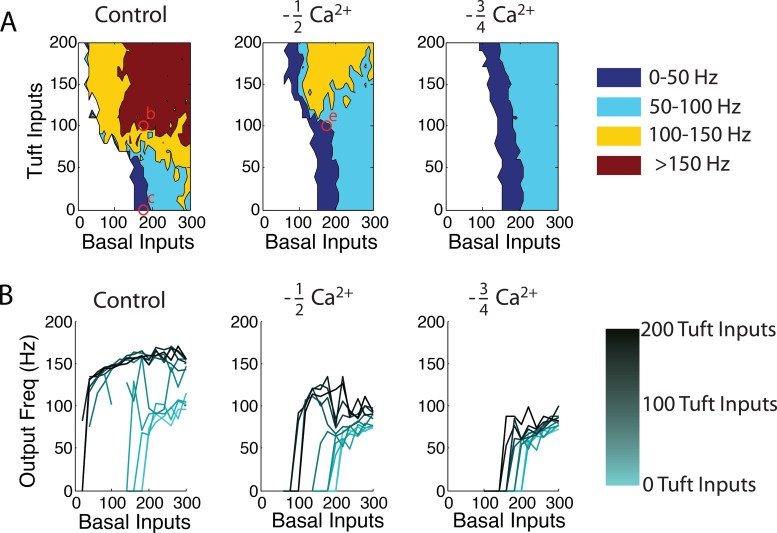
Coincidence detection details. **(a)** The output frequency of the L5 simulated cell over a wide range of tuft and basal inputs into a control cell (left), a cell with half (middle), and a quarter (right) of the Ca^2+^ conductance along the apical tuft. In each plot we systematically vary the number of basal and tuft inputs and report the output frequency at the soma in color. Open red circles correspond to Fig. 3 (B), (C), and (E). **(b)** The modulation of the input-output relationship as function of basal and apical tuft input. Different lines correspond to different amounts of tuft input (from light to dark, 0–200 tuft inputs).

We quantify the manipulation and robustness of output spike frequency by tuft inputs via simulations by varying the number of activated basal and tuft synapses. Specifically, we vary the number of tuft synapses from 0 to 200, and basal synapses from 0 to 300 per 100 ms (Fig. 4A left). We also study the impact of blocking Ca^2+^ conductances in the dendrites on tuft modulation. When Ca^2+^ conductances are reduced by 75% of the control condition (Fig. 4A right) in the apical dendrites, output frequencies above 100 Hz are abolished. At 50% of the Ca^2+^ conductances (Fig. 4A middle), frequencies from 100–150 Hz are only reached when there are (at least) 120 basal and 120 tuft inputs onto the cell. No amount of input tested resulted in >150 Hz output. The control case (Fig. 4A left) reaches outputs > 100 Hz with >80 basal and >70 tuft inputs. Spiking output of >150 Hz is reached when > 110 of basal and >90 tuft inputs, impinged on the cell. A wide range of synaptic input levels allow for tuft modulation to change somatic spiking from below to higher than 100 Hz, and, in the control case, even from no spiking to more than 100 Hz.

In Fig. 4B we show the effect of blocking Ca^2+^ conductance, which is twofold: (1) a decrease in the maximum achievable somatic spike frequency, and (2) a requirement for additional basal inputs to elicit somatic spiking for high, but not low, levels of tuft input. In both cases, this is due to the synergistic effect of somatic and dendritic depolarization. When tuft input is low, basal input alone dictates the somatic output and, thus, changes in dendritic Ca^2+^ conductance make little difference (compare light teal lines in Fig. 4B). If, instead, there is substantial tuft input (Fig. 4B dark lines), the somatic output is a function of the interaction of the soma and dendrites. That is, the somatic response for a given number of basal inputs increases substantially as dendritic Ca^2+^ conductance increases. Importantly, when tuft input increases, large increases in output spike frequency are observed. Furthermore, the extent of these increases is reduced as Ca^2+^ conductance is reduced (compare dark lines in Fig. 4B).

### A phenomenological model

**Fig 5:**
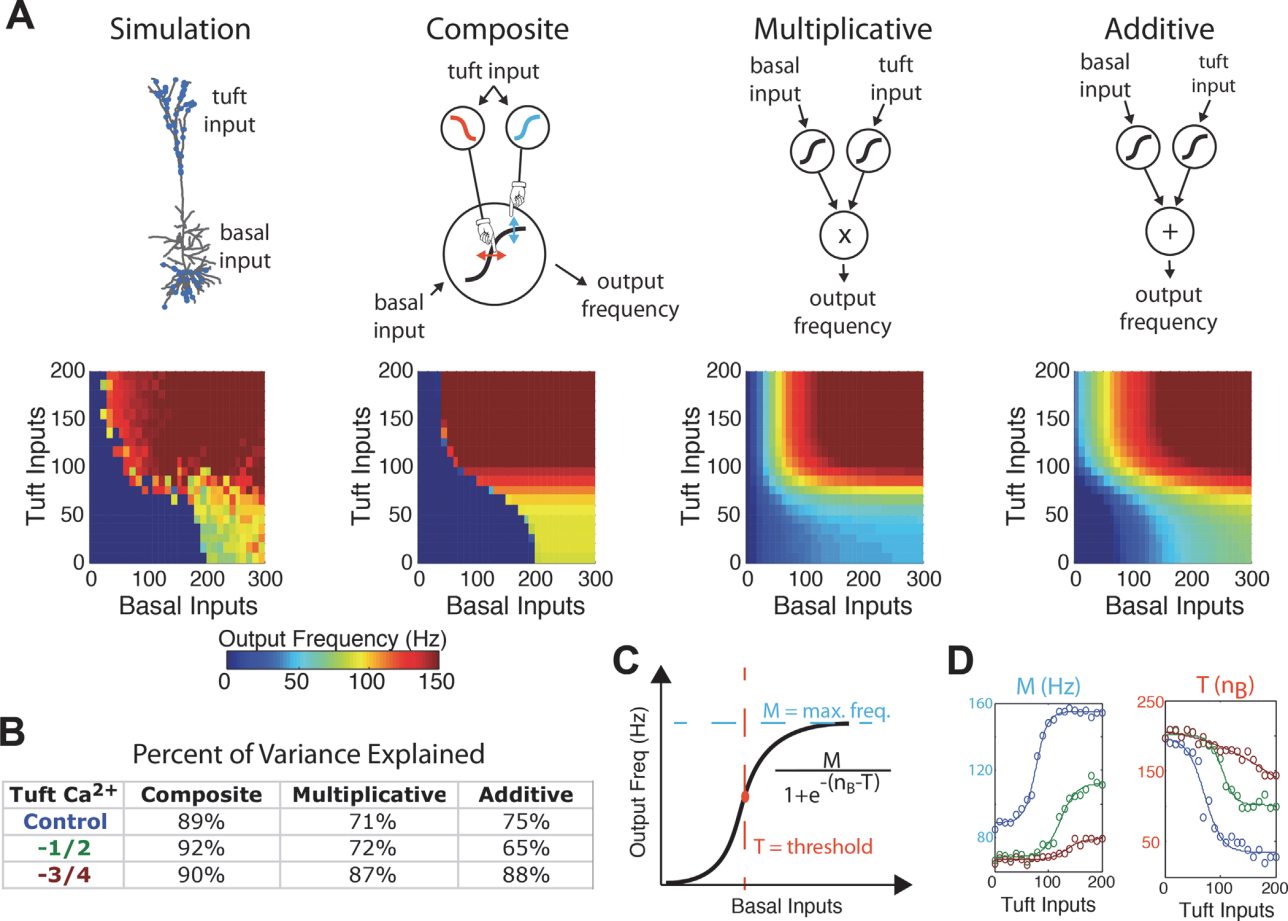
Phenomenological models. **(a)** (Top) Different phenomenological models of a L5 pyramidal cell (left to right): the detailed multi-compartmental simulation; a composite model where the maximum and threshold of the sigmoidal transformation of basal input to spike frequency are defined by tuft input; a multiplicative model which multiplies the independent sigmoidal transformations of basal and tuft output, and an additive model that adds the sigmoidal transformations of basal and tuft output. (Bottom) The output frequencies of the simulation and nonlinear least-squares best-fit models for each of the model types as a function of tuft and basal input. Note that in the composite model, the sigmoid relating tuft input to high-frequency threshold is decreasing while the sigmoid relating tuft input to maximum frequency is increasing, since tuft input acts to lower the threshold and increase the frequency of somatic output. **(b)** The percentage of variance explained of each of the three phenomenological model types. **(c)** The parameters of the composite model can be interpreted as defining the sigmoidal transformation of basal input to output frequency, where the maximum (M) and threshold (T) of that transformation is defined by the tuft input. **(d)** Plotting the maximum (left) and threshold (right) of the nonlinear least-squares fit to the simulation data (curves) agrees with tuft-constant slices of the simulation (open circles). This gives a method for interpreting and deriving the parameters of the phenomenological model. Colors refer to apical dendrite Ca^2+^ conductance amounts, as defined in (b).

To conceptually address the single-neuron computation that this biophysical setup performs, we establish a phenomenological model. We compare three models: a composite, multiplicative, and an additive model (Fig. 5A top, see Methods and S4 Fig.). Each of these models uses two sigmoidal functions to perform intermediate computations, and is justified by the existence of the two separate (one dendritic and one somatic) spiking zones in the neuron. In the composite model, basal input is transformed to output frequency via a sigmoid that has its maximum and threshold defined by tuft input (Fig. 5A, second from left). The interaction of the sigmoids in the composite model is justified by the experimental results suggesting that the result of dendritic electrogenesis is to lower the threshold for a high-frequency burst at the soma. Thus, the mathematical form used in the composite model has a dendritic sigmoid that changes the threshold and maximum firing rate of the somatic sigmoid. The multiplicative and additive models have two independent sigmoids, one of which takes tuft synapses as input, and the other which takes basal synapses as input. The result of these sigmoids is then either multiplied or summed (Fig. 5A third and fourth from the left). Importantly, the parameters of the sigmoids are left free so that the fitting process determines whether the sigmoids are increasing (e.g. the blue sigmoid in Fig. 5A) or decreasing (e.g. the red sigmoid in Fig. 5A) as a function of the independent variable. The least-squares best-fits are shown for each of the models in the bottom of Fig. 5A. The composite model outperforms both the multiplicative and additive models, though less so when Ca^2+^ conductance is decreased by 75%, suggesting that the inability of the multiplicative and additive models to represent the input-output relationship depends on dendritic electrogenesis (Fig. 5B). By directly fitting tuft-constant planes of the simulation data (horizontal planes of Fig. 5A left), to sigmoidal functions, a maximum frequency (M) and threshold (T) can be extracted for each amount of tuft input (Fig. 5C), and are shown explicitly as the open circles in Fig. 5D. The sigmoidal functions found by fitting the form of the entire composite equation to the simulation data fit the extracted M and T values well (Fig. 5D). This points to the strength of the composite model, as the same parameters can be found from the simulation data in two different ways. The effect of tuft input is to increase the maximum possible output frequency (Fig. 5D left) and decrease the threshold of basal input needed to elicit high frequency firing (Fig. 5D right). Thus, the sigmoid that relates tuft input to burst-firing threshold is decreasing (since more tuft input decreases that threshold, Fig. 5A red sigmoid), while the sigmoid that relates tuft input to somatic output frequency is increasing (since more tuft input increases the output frequency, Fig. 5A blue sigmoid). In comparison, the multiplicative and additive models only have increasing sigmoids, since the only way that these mathematical forms are able to capture the synergistic effect of tuft and basal input is to have both sigmoids at high values for increasing amounts of both tuft and basal input. Importantly, both M and T become increasingly linear as apical dendrite Ca^2+^ conductance is decreased (Fig. 5D brown lines), suggesting a reason why the simpler additive and multiplicative models perform better under those conditions (Fig. 5B). The composite model describes a coincidence detector between basal and tuft input, since only when both input streams are active in sufficient amounts is the resultant output high frequency.

### Potential mechanisms of tuning

**Fig 6:**
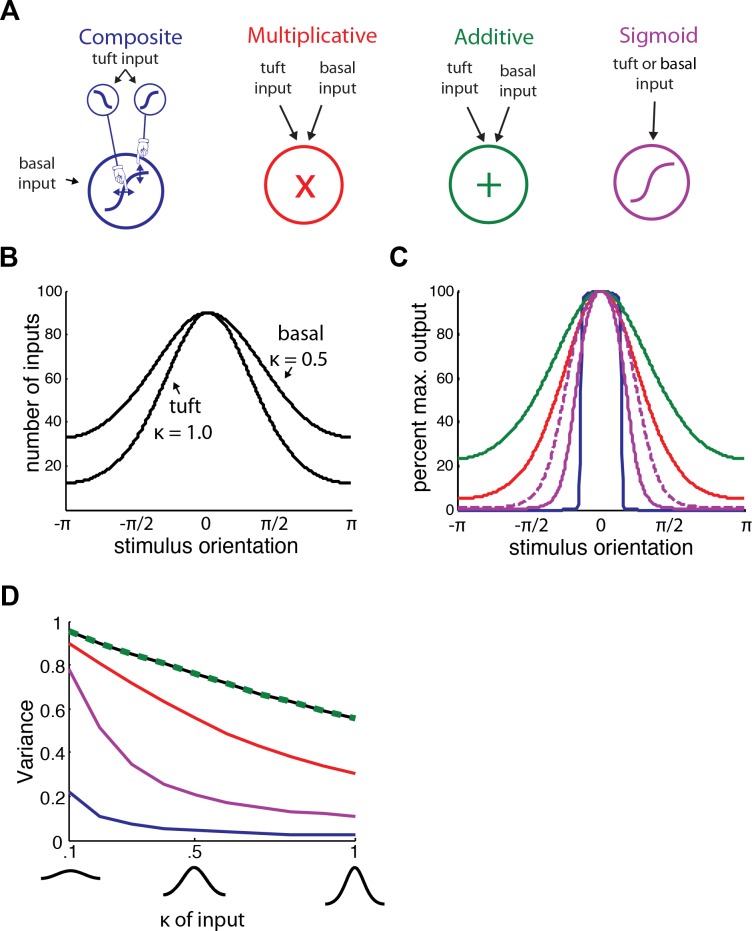
Potential mechanisms of tuning in pyramidal neurons. **(a)** Four mechanisms are compared (from left to right): composite sigmoid, purely multiplicative where the amount of tuft and basal input are simply multiplied to arrive at the final output, purely additive, where the amount of tuft and basal input are simply added to arrive at the final output, and single-sigmoid, where either the tuft or the basal input is input into a sigmoid function to arrive at the final output. **(b)** The input into these mechanisms is given by a von Mises distribution (circular analog of a normal distribution) with varying compression parameter (k) and the preferred orientation always set to 0 radians. An example of tuft and basal input distributions as a function of stimulus orientation is shown with tuft input k = 1.0 and basal input k = 0.5. **(c)** The output of the different mechanisms with the inputs shown in (b). Colors indicate the different mechanisms as defined in (a). The single-sigmoid mechanism acts on either the tuft (solid purple) or basal (dashed purple) inputs. **(d)** The circular variance of the output of the different mechanisms as a function of the width of inputs. In this plot, both tuft and basal inputs have the same k, given on the x-axis. The circular variance of the input is shown in black. Note the additive mechanism has the same output variance as the input. At all parameters of the input tested, the composite sigmoid mechanism features the tightest tuning.

How might such single cell computation be involved in visual processing? To explore tuning properties of cells employing a variety of mechanisms, we used circular distributions (von Mises distributions, see Methods and Fig. 6 and S5) to model inputs as a function of stimulus orientation. We compared four different mechanisms (Fig. 6A). A composite sigmoid as described previously, a purely multiplicative where the number of tuft and basal inputs are simply multiplied to arrive at output, a purely additive, where the number of tuft and basal inputs are simply added to arrive at output, and a single sigmoid mechanism, where either the tuft or the basal input is put through a sigmoid function to arrive at the output. The circular distributions used as inputs are normalized such that their maximums (always set to be 0 radians) are 90 synapses (Fig. 6B). When such inputs are applied to the models we see that the composite mechanism gives the tightest orientation, followed by the sigmoid, multiplicative, and additive mechanisms (Fig. 6C). This holds true for a range of input parameters (Fig. 6D).

## Discussion

Recent advances in genetic and imaging techniques have provided experimental access to aspects of cortical processing in mouse vision [18], and have, for example, given experimentalists the ability to uncover how functional maps (e.g. orientation maps) relate to long range connectivity [19,20]. However, to relate functional maps and the phenomenology of vision to computation and biophysics, it is important to know how (i.e. by what mechanism) and why (i.e. the computational role) single neurons in visual cortex respond to different types of inputs. Ultimately, we wish to describe computations that L5 pyramidal neurons perform given their input, how this supports visual perception, and to relate such computation to the biophysical details of the neuron. Understanding computation in terms of biophysics allows for direct experimental testing of network level computational hypotheses [21]. Given the unique role of L5 pyramidal neurons as one of the main integrators in the neocortical network, the electrophysiological properties of these cells hold special interest.

In this work, we focus on the conditions and mechanisms that give rise to high-frequency (>100 Hz) burst firing in L5 pyramidal neurons. Such high-frequency burst firing has been found to occur in rodent pyramidal neurons in both awake and anesthetized conditions [22]. L5 pyramidal neurons in particular have firing frequency distributions that go beyond 200 Hz, and can even have bursts of up to 6 spikes where all 6 spikes are > 100 Hz [22]. Are these bursts relevant to neural and network computation? Indeed, experimental work in the primary visual cortex of cats and monkeys has shown that bursts provide more information about the orientation or the direction of motion of visual stimulus than isolated spikes [23,24,25]. Some evidence shows that attentional effects on neurons in the visual cortex covary with burstiness [26] suggesting a top-down influence on bursting. In this work, we explored the biophysical mechanism and computation in single cells that accompanies such bursting.

Our main finding regarding single-cell physiology is that L5 pyramidal neurons of the mouse primary visual cortex (V1) broadly share the nonlinear properties of their counterparts in hippocampus, somatosensory cortex, and prefrontal cortex of mice and other mammals [2,4,5]: they support the backpropagation of APs into the dendrite, and Ca^2+^ spiking in the apical dendrites (Figs. 1, 2). Importantly, dendritic current injections farther than 250 μm from the soma are associated with long dendritic depolarizing events which precede burst firing of APs at the soma, while current injections at the soma and into the dendrite less than 250 μm from the soma are instead associated with regular trains of single APs propagating as bAPs into the dendrites (Fig. 1). Our experiments support a conception of single L5 pyramidal neurons in mouse V1 as containing two distinct areas: the perisomatic region within 250 μm of the soma and the dendritic region farther than 250 μm from the soma.

To quantify the relationship between synaptic inputs, Ca^2+^ spiking, and bAPs, we adapt a detailed biophysical multi-compartmental model able to emulate and recreate the physiological properties of mouse V1 L5 pyramids [17]. In our modeling work, we postulate two groups of excitatory, glutamatergic synaptic input. One group impinges on the basal dendrites, in the deeper layers of cortex (basal input), and the other on the apical tufts, in the upper layers (tuft input). Fig. 3 shows how tuft input changes the somatic output of a L5 pyramidal neuron from single APs (Fig. 3C) to high frequency bursts (Fig. 3B). In this way, high frequency bursts indicate the coincident input of excitatory input onto two distinct areas of the neuron. Additionally, blocking Ca^2+^ channels in the apical dendrites blocks the effect of tuft input, and reverts the neural output back to single spikes (Fig. 3E). This calcium-spike dependent modulation of output frequency by apical input can act as a mechanism for coincidence detection within individual cortical columns. In this view, coincident input into the upper and lower layers of the V1 column cause an output of a high-frequency burst (Fig. 3BC, 4). Thus, any postsynaptic cell receives a unique signal, in the form of a burst, informing of coincident input onto the presynaptic cell. Although we focus on the output of L5 pyramidal neurons, other neurons in the cortical column such as L2/3 pyramidal neurons also support Ca^2+^ dendritic electrogenesis and NMDA spiking [27], extend tuft dendrites into the upper layers, and, importantly, have long-range axons which project to other cortical areas. Here, concurrent excitatory input into the superficial and deeper layers produces high-frequency bursting, signaling coincident basal and apical input to downstream neurons. Importantly, high-frequency bursting would not occur with only basal or tuft input in isolation. Additionally, modulation of output frequency by tuft input can be vetoed by a direct block of Ca^2+^ conductance in the apical dendrite (Fig. 3 and 4), such as that produced via GABA_B_ inhibition to the distal tuft [28,29].

Further quantification of the tuft modulation, and the Ca^2+^ conductance effect on that modulation shows that it is a robust phenomenon that changes no spiking, single spikes and low-frequency spiking output to high frequency output (Fig. 4). Furthermore, blocking Ca^2+^ conductance has little effect before the nonlinear dendritic event occurs (Fig. 3FG,4). Such spiking-dependent inhibition has been referred as “silent inhibition” due to the absence of a somatic effect under subthreshold conditions [30]. Taken together, these simulations suggest a robust mechanism for coincidence detection between basal and tuft input streams. It works by changing the output frequency of the cell from low (or zero) to high firing rates.

We introduce a simple phenomenological model that reduces the coincidence detection mechanism and modulation by apical GABAergic input to the simplest possible form. A composite of sigmoids whereby input to the upper layers increases the maximum frequency and decreases the low-to-high frequency threshold of basal inputs (Fig. 5) explains the data better than more traditional multiplicative or additive models (Fig. 5B) [31,32]. It is precisely because the effect of excitatory tuft inputs is to increase the maximum frequency and decrease the threshold of the basal drive needed to elicit that maximum frequency (Fig. 4) in a Ca^2+^ spike-dependent manner that the composite model outperforms the additive and multiplicative models. In other words, the dendritic, spike-dependent manner in which tuft input changes the input-output relationship between basal input and frequency output is explicitly accounted for in the form of the composite equation. Because many aspects of the model can be interpreted in correspondence with the biophysics of the model (e.g. the maximum and threshold functions are parameterized by Ca^2+^ conductance, and the distinct inputs into the equation are distinct apical and basal excitatory synaptic pathways), this phenomenological model can be tested experimentally. Additionally, the simplicity of the model calls for it to be used in larger simulations of inter-column computation. It is important to note that while apical Ca^2+^ channels play an important role in the burst firing of pyramidal neurons [33], other ionic currents, like Na^+^ channels [34], might also contribute. The existence of such mechanisms, acting either independently or in concert with Ca^2+^ channels, could also support similar computations in pyramidal neurons.

Recently, *in vivo* work has shown that dendritic nonlinearities contribute to the orientation tuning of pyramidal neurons in the visual cortex of mice [35]. In that work, spikes were carried on long-lasting dendritic potential envelopes, such as those we observe in our *in vitro* work (Fig. 1 and 2). Additionally, hyperpolarization of the dendrites, as well as block of NMDA-mediated dendritic current (ie. the synaptic currents modeled in our work), both greatly decreased the tuning of single pyramidal neurons. In our modeling work, a decrease of synaptic input led to a loss of the coincident detection mechanism (Fig. 4). In the last part of our work we present a mechanism where dendritic spikes contribute to the orientation tuning of the cell (Fig. 6). Importantly, this mechanism is based on the biophysics we found in the *in vitro* experiments (Fig. 1 and 2) and the phenomenological model we establish (Fig. 5), and is dependent on apical tuft input (Fig. 6). For a wide range of input parameters (Figs. 6D and S6), including imprecise inputs with large variance, output variance for the composite-sigmoid model was small compared to other mechanisms.

In addition to excitatory inputs, inhibitory inputs across cortical layers are diverse. Genetically and morphologically distinct groups of interneurons contribute inhibitory inputs to specific layers of neocortex, and perform different roles [30,36]. Although no consensus has been reached about the contribution of specific inhibitory cell types to orientation tuning in mouse V1, multiple papers show that optogenetic excitation of different inhibitory cell types can influence tuning properties of nearby pyramidal neurons [37,38,39,40]. It is plausible that, like excitatory input into the apical dendrites, inhibition located in spatially distinct regions of the pyramidal neuron also contribute to orientation tuning. Layer 1 (L1) is of special interest since it contains neurogliaform cells, which release GABA nonsynaptically. Through a GABA_B_ metabotropic mechanism, the GABA ultimately causes the blockade of voltage-dependent Ca^2+^ channels, and Ca^2+^ spiking is inhibited [28]. Thus, the simulations where we reduce the conductance of voltage-gated Ca^2+^ channels in the apical dendrite can be interpreted as the physiological consequence of neurogliaform activity in L1.

In this study we found the single-cell biophysics in layer 5 pyramidal neurons supports a nonlinear coincidence detection mechanism whereby tuft input and basal input can integrate in composite-sigmoid manner. This computation can in principle explain how tuft inputs contribute to the tuning properties of pyramidal neurons in the primary visual cortex. Importantly, because the composite-sigmoid model derives closely from the biophysics of the pyramidal neuron, our results are experimentally testable. For example, electric or optogenetic manipulations of inputs onto the tuft dendrites of pyramidal neurons can be used to determine what effect those inputs have on tuning properties. Additionally, the simplicity of the phenomenological model allows it to be used in large-scale network simulations that take into account columnar structure.

## Materials and Methods

All animal work was conducted according to relevant guidelines of Berlin, Germany. Mice were anesthetized with isoflurane and then euthanized by decapitation. Experiments are performed in primary visual cortex (V1) neocortical slices from postnatal day (P)35–62 C57BL/6 mice.

### Establishing the primary visual cortex slice

Intrinsic imaging is used to localize V1 (See S1 Fig.). Visual stimuli are created using MGL, a freeware Matlab suite for psychophysics (http://gru.brain.riken.jp/doku.php/mgl/overview). Sinusoidal gratings are shown to the left eye of the mouse, positioned 20 cm from a 13 inch MacBook Pro (Apple Inc., CA) monitor. Gratings are vertically oriented or oblique (45 or 135 degrees), and have a spatial and temporal frequency of 0.045 cycles per degree and 0.069 Hz respectively [41]. Each grating is presented for 5 seconds, and is followed by 30 seconds of a black screen. Results are averaged over 30 trials. V1 (monocular) is found to be a region centered at 2.3 mm lateral from the midline, and 0.3 mm anterior to lambda, and extended at least 1 mm in the lambda direction and 1.5 mm parallel to the midline. These results agree with reported stereotaxic coordinates [42]. After intrinsic imaging, a bolus of Oregon Green Bapta is injected 500 μm below the pial surface to confirm positions of slices with respect to V1. Parasagittal slices, 300 μm thick, are taken at 12–14 degrees. L5 pyramidal neurons are patched at the soma with intracellular solution containing Alexa594 (Invitrogen) to visualize the entire dendritic tree of patched cells. The tenth or eleventh slice from the lateral edge is found to contain both the bolus injection as well as L5 apical tuft dendrites that reached the pia, confirming that we have a parallel V1 slice.

### Slice preparation

Slice preparation for electrophysiology is performed using procedures described previously [15]. Briefly, mice are decapitated and the brain is quickly removed into cold (0–4°C), oxygenated physiological solution containing (in mM), 125 NaCl, 2.5 KCl, 1.25 NaH_2_PO_4_, 25 NaHCO_3_, 1 MgCl_2_, 2 CaCl_2_, and 25 glucose, pH 7.4. Parasagittal slices, 300 μm thick, are cut from the tissue block with a vibratome (Leica VT1200S) and kept at 37°C for 30 min and then at room temperature until used.

### Electrophysiology

All experiments are performed at 32.0 ± 0.5°C. Single L5 pyramidal neurons are identified using infrared oblique illumination and a CCD camera (CoolSnap EZ; Roper Scientific). Slices are perfused with the same extracellular solution mentioned above. Recording pipettes are filled with intracellular solution containing the following (in mM): 130 K-gluconate, 5 KCl, 30 HEPES, 10 phosphocreatine, 4 MgATP, 0.3 GTP, pH 7.3. In addition, the somatic pipette contains the following: 10–50 μM Alexa 594 (Invitrogen) to visualize the dendritic arbor for dendritic patching, and 0.2% Biocytin (Sigma). Dual whole-cell voltage recordings are performed from the soma and dendrites (6–10 and 20–40 MΩ pipette resistances, respectively) using Axoclamp 2A (Molecular Devices) and Dagan BVC-700A amplifiers (Dagan Corporation). Access resistances for the dendritic recordings are 15–90 MΩ on break-through. Data is acquired with an ITC-18 board (Instrutech) and custom software written for the Igor environment (Wavemetrics).

### Data analysis

Data analysis is performed using Igor software (Wavemetrics) and Matlab (MathWorks). Statistical tests are performed with Matlab using, if not otherwise indicated, a Student's t-test in comparison of two datasets, a statistical test comparing the slope of a least-squares linear best fit line to 0, or a least-squares regression to an exponential function when such a trend is expected (subthreshold and action potential attenuation). Statistical tests are two-tailed unless a reason is explicitly stated to expect a directional relationship between two datasets.

To estimate the width of dendritic plateau potentials in the apical dendrite with long dendritic current injection, we determine the longest depolarization sustained at 20% or more above the baseline level (defined as the most hyperpolarized membrane potential during the dendritic current injection). This includes the effects of backpropagating APs as well as their interplay with the dendritic depolarization.

### Compartmental model

We modify the published Hay and colleagues [17] L5b pyramidal neuron multi-compartmental model to further probe the interaction of synaptic inputs and intrinsic membrane nonlinearities. S2 Fig. shows a table of the complete model parameters, as well as subthreshold property fits to experiment. Uniquely among multi-compartmental models, the Hay model accurately captures somatic and dendritic electrogenesis as well as the interaction between the two spiking zones (e.g. backpropagation and critical frequency). The model consists of ten active conductances, and internal Ca^2+^ dynamics to capture calcium buffering (details can be found on http://senselab.med.yale.edu/ModelDB/). Because past studies have found that dendritic electrogenesis depends on calcium channels in the apical dendrites, and that sag and dendritic resting potential and input resistance is dependent on I_h_ conductance, we were able to manipulate only dendritic calcium and I_h_ conductance parameters, by hand, to fit to our experimental results. To fit the critical frequency, we increase the dendritic low-threshold voltage-gated calcium channel conductance by a factor of 1.6. We change the I_h_ conductance to be constant in the dendritic tree, instead of exponentially increasing as a function of distance from the soma, to fit the subthreshold data (shown in S2 Fig.). The change to I_h_ conductance accounted for the differences between dendritic sag, dendritic resting membrane potential relative to the soma, and dendritic input resistance in our experiments compared to those in rat L5 somatosensory cortex (See S2 Fig.).

To add synaptic input to the model, we use an NMDA/AMPA mechanism introduced by Alon Polsky, available for download at http://senselab.med.yale.edu/modeldb and published in [3]. The NMDA conductance is voltage-dependent and given by

gNMDA(v,t)=gmaxe−t70ms−e−t3ms1+0.3e−0.08v

AMPA conductances are modeled with an instantaneous rise time and decay time constant of 0.5 ms [3]. Each synapse has a maximum NMDA to maximum AMPA conductance ratio of 1:1 (See S3 Fig.). Code to run simulations in NEURON with synapses distributed across the model (as in Fig. 3 and 4) is available for download at https://senselab.med.yale.edu/modeldb. The simulations instantiate the multicompartmental model, distribute synapses along the basal and tuft dendrites, run a 200 ms simulation, and then repeat for a wide variety of input parameters (as shown in Fig. 4).

### Phenomenological model

We create three abstract models to describe the input-output relationship from tuft and basal excitatory input to firing rate output. The three models are called additive, multiplicative, and composite. The additive and multiplicative models are composed of two sigmoids:

$$\begin{array}{l} f(n_{T})=\alpha_{1}+\frac{\alpha_{2}}{1+e^{-(n_{T}-\alpha_{3})/\alpha_{4}}}\\ g(n_{B})=\beta_{1}+\frac{\beta_{2}}{1+e^{-(n_{B}-\beta_{3})/\beta_{4}}} \end{array}$$

where n_T_ and n_B_ are the number of tuft and basal inputs, respectively, and all α and ßs are free parameters. In the additive and multiplicative models, the tuft and basal sigmoids are either added or multiplied to arrive at the final output frequency:

$$\begin{array}{l} freq_{add}=f(n_{T})+g(n_{B})\\ freq_{mult}=f(n_{T})g(n_{B}) \end{array}$$

The composite model is similarly made up of two sigmoids, both functions of the tuft input:

$$\begin{array}{l} M\text{​}(n_{T})=\alpha_{1}+\frac{\alpha_{2}}{1+e^{-(n_{T}-\alpha_{3})/\alpha_{4}}}\\ T(n_{T})=\beta_{1}+\frac{\beta_{2}}{1+e^{-(n_{T}-\beta_{3})/\beta_{4}}} \end{array}$$

The final output frequency is then given by:

$$freq_{composite}(n_{B})=\frac{M(n_{T})}{1+e^{-(n_{B}-T(n_{T}))}}$$

Such that maximum and threshold of the basal input sigmoid are defined by the tuft input functions, *M(n*_*T*_) and *T(n*_*T*_). Code containing all three phenomenological models, including their parameters, is available at https://senselab.med.yale.edu/modeldb.

### Mechanistic models

To explore potential mechanisms of tuning in pyramidal neurons with both basal and tuft inputs, we described inputs as a function the orientation of a visual input (S5 Fig.). The number of inputs was given by a von Mises distribution that was normalized such that at the maximum orientation, the number of synapses was 90. Every von Mises distribution has two parameters, a preferred orientation (here always set to 0 radians), and a compression factor κ (with 1/ κ being the circular analog to variance), ranging from 0.1 to 1. We compare the composite sigmoid mechanism to a purely additive (simply adding the two distributions), a purely multiplicative (simply multiplying the two distributions), and a single-sigmoid mechanism. Since the single sigmoid mechanism only takes one input, we assign the parameters to be optimized for a maximum of 90 inputs, as shown in S5 Fig.. Compared outputs are always normalized to the maximum output generated by the mechanism.

## Supporting Information

S1 Fig Intrinsic imaging to find a parallel V1 slice. In order to perform this study, we first established a V1, 300 micron slice, which had the full dendritic extent of L5 pyramidal neuron dendrites (ie. The slice had to be normal to the brain surface). To find V1, we used intrinsic imaging with visual stimulus (details in Materials and Methods). **a,** Image of the craniotomy used during the intrinsic imaging experiment. **b,** the difference in intrinsic signal between visual stimulus and darkness conditions. **c,** Allen Brain Reference Atlas sagittal section with visual cortex in green. **d,** Brightfield image of the V1 slice. **e,** Fluorescence signal of injected Oregon Green BAPTA bolus into the V1 area found in the intrinsic imaging experiment, verifying that the slice contains V1. **f,** Biocytin stain of a L5 pyramidal neuron in the V1 slice, verifying that we have a V1 slice with the entire extent of the L5 dendritic tree.
(PNG)

S2 Fig The computational model. **(a)** Diagram of the computational model colored by section name. **(b)** The difference in resting membrane potential of dendrite and soma, sag, and input resistance as a function of distance from the soma in experiments (black) and the model (blue). **(c)** The computational model parameters. Note that a voltage-gated calcium conductance was increased 100-fold in a 200 micron area around the main bifurcation point to model the calcium hotzone (see Hay et al. (2011)), and the exponential increase of Ih conductance was replaced with a flat Ih conductance spatial profile along the main dendritic axis.
(PNG)

S3 Fig Layer 5 pyramidal neurons in mouse V1 and the computational model have NMDA dependent nonlinearities that produce NMDA spikes in the dendrites. (a) Diagram of the experimental setup. A glass theta pipette (green) is used for local extracellular stimulation, while a somatic whole cell patch (yellow) recorded in current clamp at the soma. (b) Two short extracellular pulses at 50 Hz elicited two EPSPs at the soma. In control cases (black), a sharp nonlinear increase in both the duration and amplitude of the second EPSP occurs past some threshold. Bath application of AP-5 (red), an NMDAr antagonist, eliminates this nonlinear effect. (c) The integral of the second EPSP as a function of extracellular stimulus intensity. Note the sharp nonlinear increase in the integral past some threshold only in the control case. (d) Summary of the extracellular stimulation for 6 cells. Left, subthreshold comparison of AP-5 (red circles) and control (black circles) integrals of the second EPSP. Lines connecting circles indicate pairs from the same cell and extracellular stimulus location. The difference between AP-5 and control conditions for the subthreshold case is insignificant. Right, suprathreshold comparison of AP-5 and control integrals of the second EPSP. Control suprathreshold EPSP integrals are significantly bigger than under AP-5 conditions (p<0.01). (e) diagram of the location of the synapse (green) in the simulation. (top) The somatic membrane potential with (black) and without (red) NMDA conductance, in response to increasing synaptic conductance. (bottom) The membrane potential at the location of the synapse.
(PNG)

S4 Fig Phenomenological model outputs. Shown are the output frequencies as a function of tuft and basal inputs into the simulated morphological neuron, and the three tested phenomenological models, during control and reduced Ca^2+^ conductance conditions.
(PNG)

S5 Fig Details of tuning mechanisms. **(a)** The sigmoid functions used for the composite (blue) and single sigmoid (purple) mechanisms. **(b)** The number of inputs is defined by a von Mises distribution always centered at 0 radians normalized such that the maximum number of inputs is 90. The only parameter varied for the input is the compression parameter k (where 1/k is the circular analog to the variance of a normal distribution). The output variance of the four different mechanisms is given in **(c-f)**.
(PNG)
