# Supplementary figures and images for: Physiology of Layer 5 Pyramidal Neurons in Mouse Primary Visual Cortex: Coincidence Detection through Bursting
Source: PLoS Comput Biol. 2015 Mar 13;11(3):e1004090. doi: 10.1371/journal.pcbi.1004090 (PMC4358988; doi:10.1371/journal.pcbi.1004090)

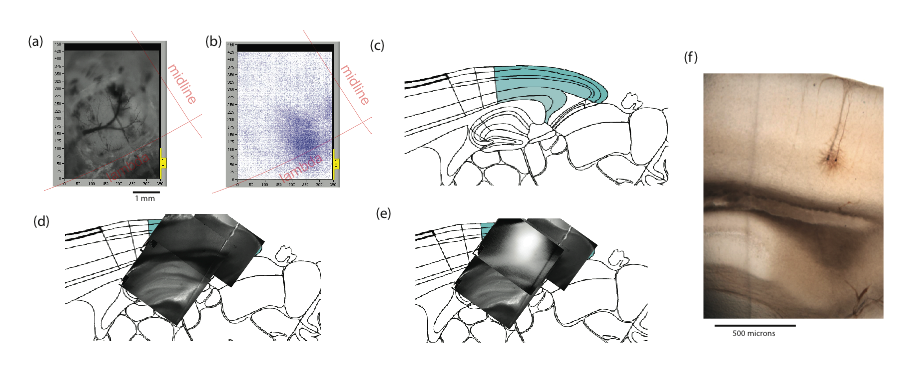

Supplement: S1 Fig — In order to perform this study, we first established a V1, 300 micron slice, which had the full dendritic extent of L5 pyramidal neuron dendrites (ie. The slice had to be normal to the brain surface). To find V1, we used intrinsic imaging with visual stimulus (details in Materials and Methods). a, Image of the craniotomy used during the intrinsic imaging experiment. b, the difference in intrinsic signal between visual stimulus and darkness conditions. c, Allen Brain Reference Atlas sagittal section with visual cortex in green. d, Brightfield image of the V1 slice. e, Fluorescence signal of injected Oregon Green BAPTA bolus into the V1 area found in the intrinsic imaging experiment, verifying that the slice contains V1. f, Biocytin stain of a L5 pyramidal neuron in the V1 slice, verifying that we have a V1 slice with the entire extent of the L5 dendritic tree. (PNG) [file pcbi.1004090.s001.png]

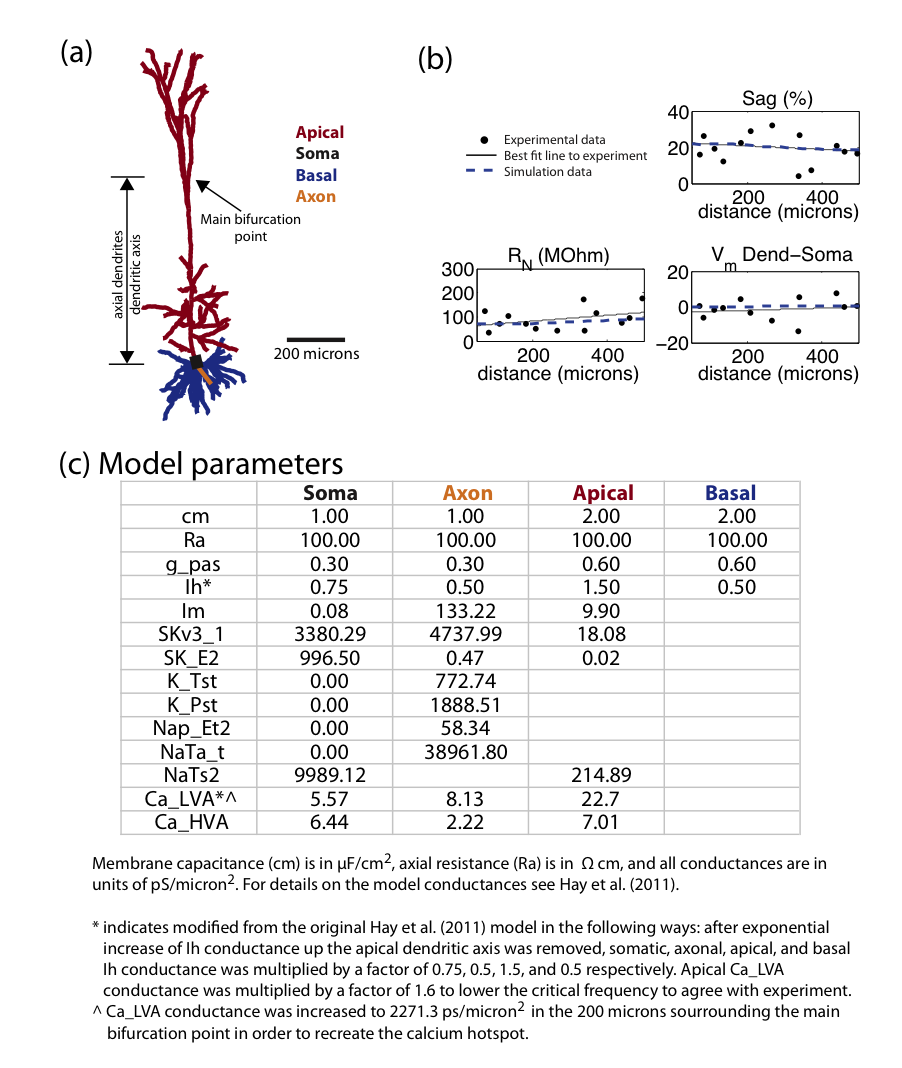

Supplement: S2 Fig — (a) Diagram of the computational model colored by section name. (b) The difference in resting membrane potential of dendrite and soma, sag, and input resistance as a function of distance from the soma in experiments (black) and the model (blue). (c) The computational model parameters. Note that a voltage-gated calcium conductance was increased 100-fold in a 200 micron area around the main bifurcation point to model the calcium hotzone (see Hay et al. (2011)), and the exponential increase of Ih conductance was replaced with a flat Ih conductance spatial profile along the main dendritic axis. (PNG) [file pcbi.1004090.s002.png]

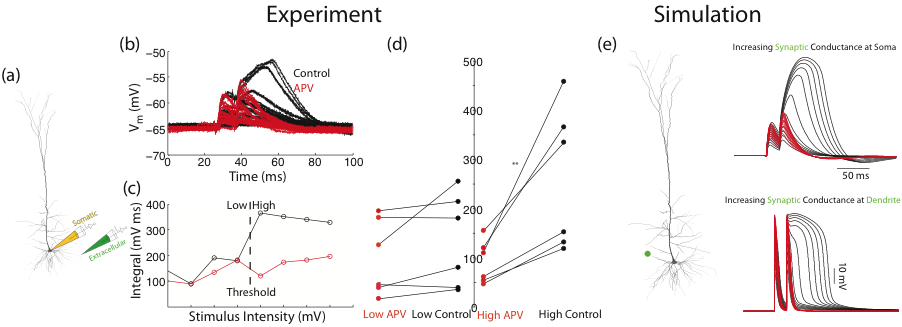

Supplement: S3 Fig — (a) Diagram of the experimental setup. A glass theta pipette (green) is used for local extracellular stimulation, while a somatic whole cell patch (yellow) recorded in current clamp at the soma. (b) Two short extracellular pulses at 50 Hz elicited two EPSPs at the soma. In control cases (black), a sharp nonlinear increase in both the duration and amplitude of the second EPSP occurs past some threshold. Bath application of AP-5 (red), an NMDAr antagonist, eliminates this nonlinear effect. (c) The integral of the second EPSP as a function of extracellular stimulus intensity. Note the sharp nonlinear increase in the integral past some threshold only in the control case. (d) Summary of the extracellular stimulation for 6 cells. Left, subthreshold comparison of AP-5 (red circles) and control (black circles) integrals of the second EPSP. Lines connecting circles indicate pairs from the same cell and extracellular stimulus location. The difference between AP-5 and control conditions for the subthreshold case is insignificant. Right, suprathreshold comparison of AP-5 and control integrals of the second EPSP. Control suprathreshold EPSP integrals are significantly bigger than under AP-5 conditions (p<0.01). (e) diagram of the location of the synapse (green) in the simulation. (top) The somatic membrane potential with (black) and without (red) NMDA conductance, in response to increasing synaptic conductance. (bottom) The membrane potential at the location of the synapse. (PNG) [file pcbi.1004090.s003.png]

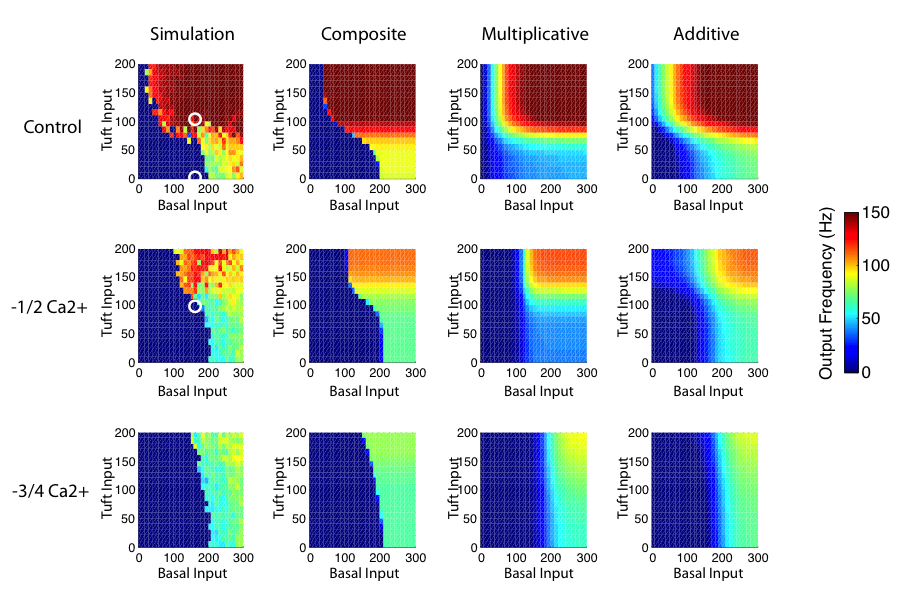

Supplement: S4 Fig — Shown are the output frequencies as a function of tuft and basal inputs into the simulated morphological neuron, and the three tested phenomenological models, during control and reduced Ca2+ conductance conditions. (PNG) [file pcbi.1004090.s004.png]

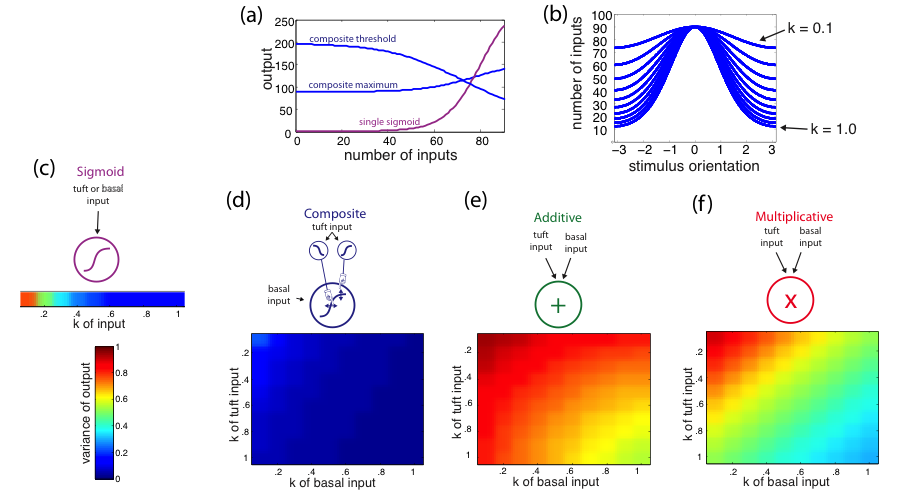

Supplement: S5 Fig — (a) The sigmoid functions used for the composite (blue) and single sigmoid (purple) mechanisms. (b) The number of inputs is defined by a von Mises distribution always centered at 0 radians normalized such that the maximum number of inputs is 90. The only parameter varied for the input is the compression parameter k (where 1/k is the circular analog to the variance of a normal distribution). The output variance of the four different mechanisms is given in (c-f). (PNG) [file pcbi.1004090.s005.png]
